# Caries of a Molar Tooth of the Superior Maxilla, with Necrosis of the Jaws

**Published:** 1882-10

**Authors:** Frank V. Walton


New York City, August, 1882.

### VII.—CARIES OF A MOLAR TOOTH OF THE SUPERIOR MAXILLA WITH NECROSIS OF THE JAW.

REPORTED BY FRANK V. WALTON, D.V.S.

Case, a young gelding, æt. 6. Was bought January 6th, 1882. At the time of purchasing a slight swelling was noticed upon the left superior maxillary bone. At the centre of this swelling there was a slight brake in the skin, with a small discharge of pus. The purchaser took this to be a bruise, with slight suppuration, probably from an injury caused by transportation in the cars.

After getting the horse to his stable it was noticed that the tumor rapidly enlarged, the discharge became more profuse, and had a very offensive odor.

At the first examination a probe easily passed from the external opening through the alveolar process of the upper jaw, and on into the mouth. At this time a few small fragments of necrosed bone were detected.

A diagnosis of necrosis of the jaw with ulceration of one of the teeth was made. A removal of the affected tooth and the necrosed bone was advised, which was wisely consented to by the owners.

An attempt was made to remove the tooth with the dental forceps commonly used in veterinary practice. It was found to be an impossibility to draw the tooth with the power afforded by such an instrument. It was ascertained that the crown of the tooth could be easily crushed off by the instrument; but this would not reach the original difficulty. All attempts in this line were then abandoned.

The following day the horse was thrown,secured, and ether administered. A crucial incision was made by Prof. Porter over the most prominent portion of the enlargement,the four flaps turned back, and the teeth and superior maxilla fully exposed. Having accomplished this, the external alveolar process was chiselled away, exposing the root of the tooth for its whole length. The necrosed bone was also removed. It was now quite an easy matter to pry out the affected tooth. There was quite a profuse capillary hemorrhage for a few minutes; this, however, soon stopped upon the application of ice-cold water.

The horse quickly rose to his feet and seemed to be greatly relieved.

The external half of the incisions was closed with sutures, but the centre was left open so that the wound could be more easily cleansed.

The wound was thoroughly washed with a carbolic acid solution, and then injected with a solution of copper sulphate, ten grains to one ounce of water.

The animal made a speedy recovery, took to his feed well the next day, and in two weeks was at work and feeling quite well. The external opening was not allowed to close for some time, so that in case there were any small fragments of necrosed bone to come away, they could be more easily manipulated.

One remarkable feature in this case was the fact that there was no offensive odor from the wound, and the horse took to his feed the next day, and rapidly gained in general health and spirits.

Microscopical examination showed that there was nothing malignant in this trouble.

New York City, August, 1882.

[This case shows the efficacy of copper solutions in the treatment of cavious and necrosed bone.

This case ought to be one of great interest to the veterinary surgeon, for tumors of the superior maxilla are not infrequent, and many of them prove to be quite malignant in character.

This, in connection with similar cases, goes to show that it is reasonable to suppose that many of them commence in this simple manner. Caries of the root of the tooth and slight necrosis, which, if not speedily removed, acts as a constant irritant, often working its way into the maxillary sinous, and in the long run producing a malignant and incurable disease.

The ease with which the operation was performed and the rapid and complete recovery of the case are strong arguments in favor of such operations being done early and completely.

In most of these cases we think it would be much better, instead of making a crucial incision, to slit up the lip toward the ear and turn up the upper lip. In this way the jaw could be quite as easily reached, and there would be the least possible deformity after recovery.—Ed.]

